# Detection of hepatocellular carcinoma feeding vessels: MDCT angiography with 3D reconstruction versus digital subtraction angiography

**DOI:** 10.1186/s12880-024-01408-z

**Published:** 2024-09-18

**Authors:** Ramy M. Ahmed, Wageeh A. Ali, Ahmed M. AbdelHakam, Sayed H. Ahmed

**Affiliations:** Department of Radiology, Faculty of Medicine, Assiut University, Assiut, Egypt

**Keywords:** HCC, TACE, Feeding arteries, 3DCT, 3D, VR, DSA.

## Abstract

**Background:**

Accurate detection of Hepatocellular carcinoma (HCC) feeding vessels during transcatheter arterial chemoembolization (TACE) is important for an effective treatment, while limiting non-target embolization. This study aimed to investigate the feasibility and accuracy of pre-TACE three dimensional (3D) CT angiography for tumor-feeding vessels detection compared to DSA.

**Methods:**

Sixty-nine consecutive patients referred for TACE from May 2022 to May 2023 were included. (3D) CT images were reconstructed from the pre-TACE diagnostic multiphasic contrast enhanced CT images and compared with non-selective digital subtraction angiography (DSA) images obtained during TACE for detection of HCC feeding vessels. A “Ground truth” made by consensus between observers after reviewing all available pre-TACE CT images, and DSA and CBCT images during TACE to detect the true feeding vessels was the gold standard. Sensitivity, specificity, negative predictive value (NPV), positive predictive value (PPV), accuracy and ROC curve with AUC were calculated for each modality and compared.

**Results:**

A total of 136 active HCCs were detected in the 69 consecutive patients included in the study. 185 feeding arteries were detected by 3D CT and DSA and included in the analysis. 3D CT detection of feeding arteries revealed mean sensitivity, specificity, PPV, NPV and accuracy of 91%, 71%, 98%, 36%, and 90%, respectively, with mean AUC = 0.81. DSA detection of feeding arteries revealed mean sensitivity, specificity, PPV, NPV, and accuracy of 80%, 58%, 96.5%, 16.5% and 78%, respectively, with mean AUC = 0.69.

**Conclusions:**

Pre-TACE 3D CT angiography has shown promise in improving the detection of HCC feeding vessels compared to DSA. However, further studies are required to confirm these findings across different clinical settings and patient populations.

**Trial registration:**

This study was prospectively registered at Clinicaltrials.gov with ID NCT05304572; Date of registration: 2-4-2022.

**Supplementary Information:**

The online version contains supplementary material available at 10.1186/s12880-024-01408-z.

## Background

Hepatocellular carcinoma (HCC) ranks as the sixth most prevalent cancer globally [1]. For patients with intermediate-stage HCC, transcatheter arterial chemoembolization (TACE) serves as a palliative treatment [2]. This approach enables the targeted delivery of potent chemotherapeutic agents to the tumor, inducing ischemic cell death [3]. To ensure effective treatment while minimizing unintended embolization, it is crucial to accurately identify the vessels supplying the tumor. Traditionally, digital subtraction angiography (DSA) has been employed for detecting these feeding vessels. However, the overlapping of vessels can lead to misinterpretation, necessitating multiple injections and projections, thereby prolonging procedural time, increasing contrast dose, and enhancing radiation exposure [4].

A more recent technique, known as three-dimensional (3D) cone-beam computed tomography (CB-CT) angiography, offers a comprehensive visualization of the vascular anatomy following a single contrast injection into the hepatic artery [5, 6]. Nonetheless, the adoption of this method for routine use may be hindered by factors such as limited accessibility, concerns over radiation exposure, and the time required for data processing. In an effort to address these challenges, novel software programs have been developed to automatically detect feeding vessels in CB-CT scans. However, their widespread availability remains limited [7, 8].

Multiphasic contrast-enhanced CT is one of the recommended imaging tools for the diagnosis of HCC and is routinely performed before TACE [9]. 3D CT angiographic images can be reconstructed from arterial phase images and help in the detection of HCC-feeding vessels.

To enhance catheter navigation during TACE, a 2D-3D registration technique integrating pre/perioperative 3D vasculature data with intraoperative 2D X-ray images is proposed, offering a real-time roadmap to improve procedural guidance and reduce intervention time, radiation exposure, and contrast agent use. However, it can be computationally intensive, requiring significant processing power and time and not widely available [10].

However, there are few reports on the application of Pre-TACE 3D CT angiography images in planning TACE procedures [11, 12]. This study therefore aimed to investigate the feasibility and accuracy of Pre-TACE 3D CT angiography for detecting tumor-feeding vessels compared to DSA.

## Materials and methods

This was a prospective observational cross-sectional study conducted on consecutive patients from May 2022 to May 2023 who were referred to our department for TACE; the inclusion criteria included very early, early or intermediate stage HCC patients according to the Barcelona Clinic Liver Cancer staging system (BCLC) [13] with the following criteria; HCC not suitable for resection, liver transplantation, or percutaneous ablation with underlying CHILD class A/B cirrhosis, a patent main portal vein, less than 50% involvement of the liver by the tumor, no vascular invasion or extrahepatic tumor spread, normal renal function and a bilirubin level < 2 mg/dl. Informed written consent was obtained from all patients. The study protocol was approved by the Medical Ethical Committee of our institution (IRB.no. 17300765).

### 3D CT angiography

DICOM images from a recent multiphasic CT examination including at least the arterial and portal phases – either inside or outside our hospital – performed within 4 weeks interval [14] before TACE for HCC diagnosis were obtained for analysis. Patients with unavailable CT raw DICOM images or patients with only available MR images were excluded.

Multiphasic CT in our hospital was routinely performed on a 16-detector CT machine (SOMATOM, Siemens Healthcare, Germany) including non-enhanced, early arterial, late arterial, portal and equilibrium phases with the following scanning parameters: 120 kV, 180 MAs, a beam collimation of 0.75 mm, and a helical pitch of 1.15 mm. A 75-100-mL bolus of iodinated contrast material (Omnipaque 350 mg I/mL, GE Healthcare, 1.5 mL/kg of body weight, up to a maximum of 150 mL) was administered at a rate of 3–4 mL/s. Data acquisition for the early arterial phase was initiated immediately after a threshold level of 100 HU was reached in the abdominal aorta. The late arterial phase was obtained 4 s after the end of the early arterial phase in the caudo-cranial direction. Portal phase images were obtained 12 s after the end of the late arterial phase, and equilibrium phase images were obtained 120 s later. The parameters of the CT scans conducted outside our institution differed from ours in terms of the quantity and concentration of contrast agent used, specifically 70–80 ml of iodinated contrast material (Omnipaque 300 mg I/mL, GE Healthcare), and the absence of late arterial phase acquisition.

The source images of the arterial and portal phases were transferred to the workstation (GE, Advantage 4.6) to produce 3D volume rendering (VR) angiographic images as follows: (a) a background 3D VR image of the skeleton was obtained and stored as a separate image; (b) a separate 3D VR angiographic image of the contrast opacified aorta and its main abdominal branches in the early arterial phase was obtained, and then all intrahepatic branches were traced manually by the operator at axial images and added to the 3D VR angiographic image; and (c) a separate 3D VR image of the tumor was obtained by manual selection of the tumor and a surrounding margin of approximately 1 cm on the axial images of either the arterial phase (early contrast-enhanced area) or from the portal phase (contrast-washed area). Finally, all these images were merged and saved in various degrees of rotation with and without bone background images to facilitate the detection of feeding arteries of the tumor by observers (See supplementary materials 1, 2 and 3).

### DSA during TACE

TACE was performed using a DSA machine (Artis Zeego Q, VE 40 A, Siemens, Germany) with 3 frames/sec vascular protocol. After percutaneous common femoral artery access, a 5Fr-Cobra (C2) or sidewinder (SIM1) catheter was used for catheterizing the celiac trunk and superior mesenteric artery (SMA). Celiac and SMA angiographies were performed by injecting 20 mL of 350 mg/mL Omnipaque via the forced manual injection method for 4–6 s. Selective DSA of the common hepatic artery (CHA), hepatic artery proper (PHA) or replaced hepatic arteries was performed through a 5Fr catheter (15 mL Omnipaque) or through a coaxially placed 2.7Fr microcatheter (Progreat; Terumo) with 5 mL Omnipaque if the 5Fr catheter could not be advanced into these arteries. Then, a microcatheter was used for selective access of the feeding hepatic arteries as close as possible to the tumor to inject chemotherapeutic drug (Doxorubicin 50 mg) through the feeding artery and then embolized using a gelatine sponge.

### Image analysis and ground truth

Tumor size was measured as the largest tumor diameter determined on transverse CT images and then subdivided into two groups: ***(a) HCC > 2*** cm or ***(b) HCC ≤*** 2 cm. HCC lesions were categorized as ***(a) encapsulated HCC*** (predominantly a round lesion with the presence of a capsule) or ***(b) diffuse HCC*** (predominantly irregular or lobular lesions without a capsule).

3D CT and DSA image quality was rated as follows: ***(a) subsegmental***: clear visualization of all hepatic arteries, including subsegmental arteries; ***(b) segmental***: clear visualization of hepatic arteries up to the segmental level; ***(c) lobar***: clear visualization of hepatic arteries up to the lobar level; and ***(d) blurred***: blurring of hepatic arteries with difficulty in tracing them. All patients with blurred images in either modality were excluded.

Two radiologists experienced in hepatic imaging and abdominal intervention (10- and 15-years’ experience) independently viewed the DSA and 3D CT images for the detection of hepatic arterial variant anatomy, HCC lesions and their feeding arteries. The observers evaluated the DSA images of the celiac, SMA, CHA, PHA or replaced hepatic arteries obtained during TACE but were blinded to any additional selective DSA of the segmental arteries and feeding arteries. The DSA images were initially interpreted, and then 3D CT images were interpreted at least 2 weeks later to minimize memory bias.

Each observer was asked to record all possible feeding arteries of each lesion on 3D CT images and DSA images with a confidence score as follows: ***(1) definitely a feeding artery*** (evident connection to the tumor), ***(2) probably a feeding artery*** (possible connection to the tumor), ***(3) intermediate probability*** (no visible connection to the tumor but it is the nearest possible artery supplying the lesion area), and ***(4) no detectable feeding artery***.

**Fig. 1:**
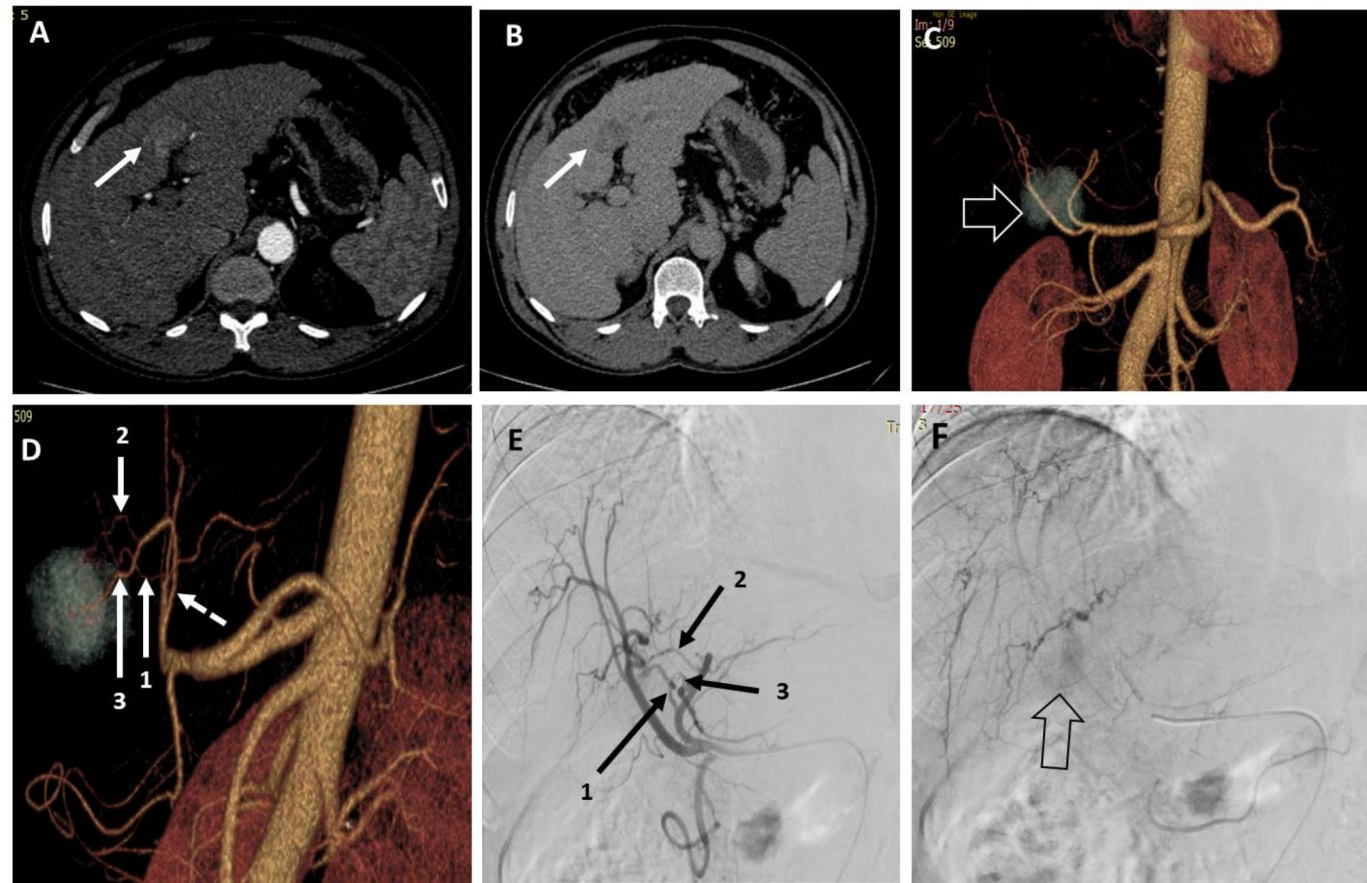
A patient with segment III-IV HCC measuring 3.5 × 3.3 cm. (**A**) and (**B**) are axial CT images in the arterial and venous phases, respectively, showing arterial postcontrast enhancement of the lesion with washout in the venous phase. (**C**) 3D-reconstructed AP image showing the lesion (hollow arrow) located behind the bifurcation of the proper hepatic artery. (**D**) 3D-reconstructed image in oblique view showing three possible feeding branches to the lesion arising from the left hepatic artery (dashed artery). (**E**) DSA of the CHA using a 5 Fr catheter showing three possible feeding arteries arising from the left hepatic artery. (**F**) Late-phase image during DSA of the CHA showing faint contrast blush of the lesion (hollow arrow). Artery 1 was identified by both observers via 3D and DSA images. Artery 2 was identified by both observers via 3D and DSA images. Artery 3 was identified by both observers in 3D images but could not be identified in DSA images. The three arteries are true feeding vessels according to the ground truth

**The “ground truth” (GT)** was the gold standard for identifying true feeding arteries. It was made by a consensus among all radiologists participating in the study after reviewing: (a) pre-TACE multiphasic CT MPR, MIP and 3D images, (b) all acquired DSA images during TACE including nonselective, selective, segmental and feeding artery angiograms before chemo-embolizing agent injection, (c) contrast-enhanced CB-CT images if available and (d) post-lipiodol injection CB-CT images if available. Based on the ground truth, the suspected feeding arteries identified by both observers in the 3D CT and DSA images were categorized as ***true positive (TP)***,*** false positive (FP)***,*** true negative (TN) or false negative (FN)*** (Fig. 1).

### Statistical analysis

The sensitivity, specificity, positive predictive value (PPV), negative predictive value (NPV) and accuracy of each modality were calculated, and ROC analysis was performed for each observer and each modality. The diagnostic performance of the two imaging modalities was compared using the area under the ROC curve (AUC) for each technique for the same observer. Interobserver agreement was assessed by using the Cohen’s kappa test for each modality a Chi-Square test (X^2^) was used to detect associations between categorical variables, with Post-hoc analysis using Bonferroni test on adjusted residuals. Data processing and analysis were performed using SPSS version 21.0 (SPSS, Inc., IBM Company, Chicago, IL). Two-tailed P values < 0.05 were considered to indicate statistical significance.

## Results

Sixty-nine consecutive patients were included in the study; 59 were males (85.5%), and 10 were females (14.5%), with a mean age of 65 ± 6 years (range, 54–81 years). Forty-four patients (63.8%) had their CT examinations conducted at our institution, while the remaining 25 patients underwent CT scans at four other hospitals.

A total of 136 active HCCs were detected among the 69 patients included in the study. The mean size of the 136 included active lesions was 4.2 cm ± 3.6 cm (median/interquartile range (IQR) = 3.3/3.8 cm), and the lesions ranged in size from 1 to 24 cm. Ninety-six lesions (70.6%) were > 2 cm in size, and 40 lesions (29.4%) were ≤ 2 cm in size. A total of 129 lesions (94.9%) were capsulated, and the remaining 7 lesions (5.1%) were diffuse.

The 3D CT versus DSA quality grades were subsegmental in 47 (68.1%) versus 50 patients (72.5%), segmental in 21 (30.4%) versus 18 (26.1%), and lobar in one (1.4%) versus one (1.4%) patient, respectively, with no statistically significant difference between them (chi-square test (X2), *P* = 0.9).

**Fig. 2:**
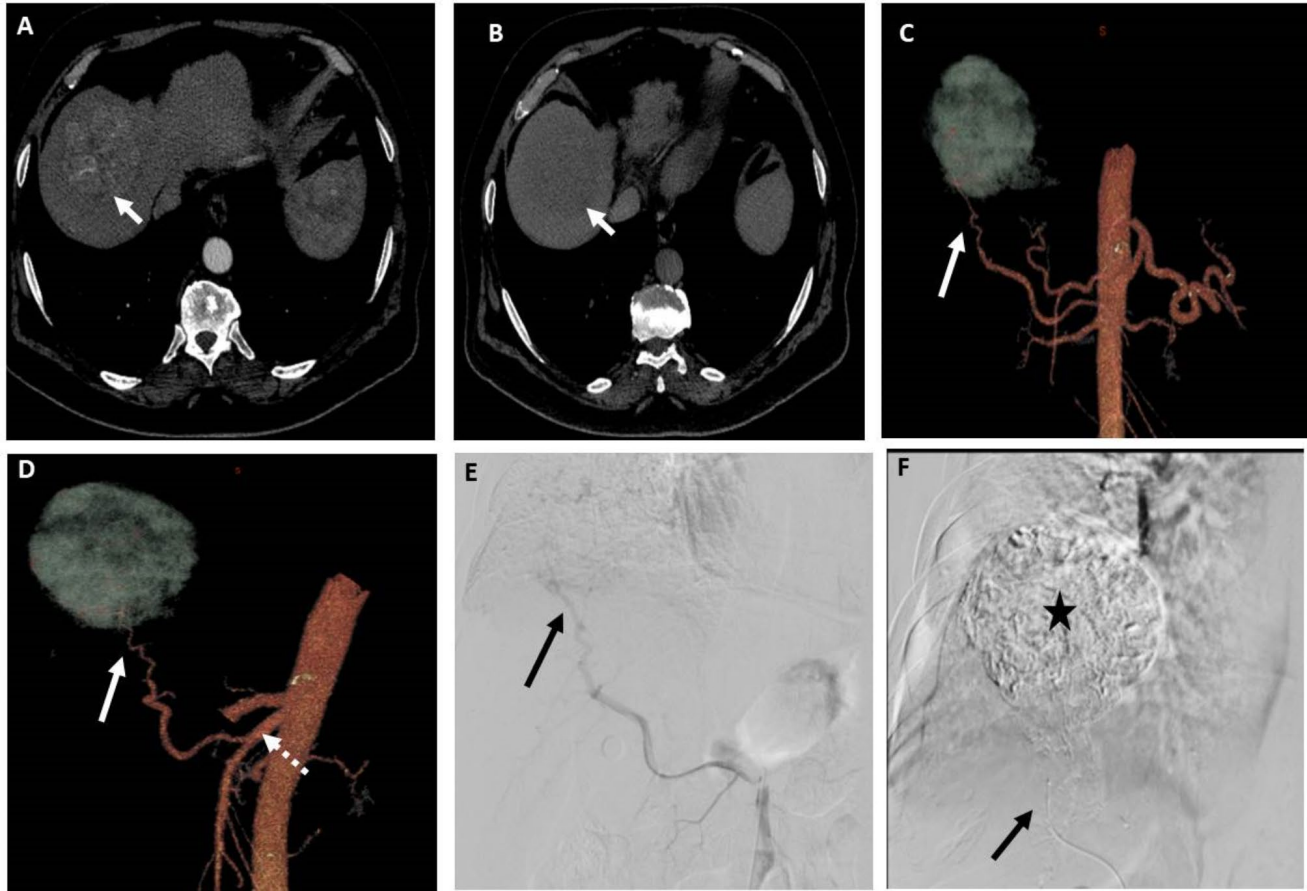
A patient with segment VII HCC measuring 6.5 cm. (**A**) and (**B**) Axial CT images in the arterial and venous phases, respectively, showing early arterial contrast enhancement of the lesion and contrast washout in the venous phase relative to the surrounding liver. (**C**) and (**D**) 3D reconstructed CT angiography images in AP view and oblique views, respectively, showing the possible feeding artery (arrow) to the lesion arising from replaced right hepatic artery from SMA (dashed arrow). (**E**) DSA of the SMA at the origin of the replaced right hepatic artery using 5Fr catheter showing the possible feeding artery (arrow). (**F**) Post-TACE DSA image showing complete opacification of the lesion with lipiodol after chemotherapy-lipiodol mixture injection through the microcatheter (arrow) in the feeding artery. Both observers identified the feeding artery in 3D images and DSA images

Hepatic arterial variant anatomy was noted in 28 patients (40.5%); replaced right hepatic artery from the SMA in 20 patients (Fig. 2), accessory left hepatic artery in three patients, replaced left hepatic artery arising from the left gastric artery in one patient, middle hepatic artery arising from the gastroduodenal artery in one patient, CHA arising from the SMA in one patient, CHA arising from the aorta in one patient, and separate origin of both segment III and II arteries from the CHA in one patient. One patient had occlusion of the celiac trunk origin, and another patient had celiac stenosis with prominent pancreaticoduodenal collateral arteries in both patients. DSA and 3D CT image findings were consistent in 100% of these patients according to both observers.

### Feeding artery detection (Table 1)

A total of 185 feeding arteries were detected by 3D CT and DSA by both observers and included in the analysis; 173 of them (93.5%) were true feeding arteries according to the ground truth. 3D CT detection of feeding arteries by observer1 versus observer2 revealed a sensitivity of 91.9% versus 90.8% (mean = 91%), a specificity of 66.7% versus 75% (mean = 71%), a PPV of 97.5% versus 98.1% (mean = 98%), an NPV of 36.4% versus 36% (mean = 36%), and an accuracy of 90.3% versus 89.7% (mean = 90%). ROC curve analysis of the results for observers 1 and 2 revealed that the AUC was 0.79 (0.63–0.96, 95% CI; *P* = 0.001) and 0.83 (0.68–0.98, 95% CI; *P* = 0.0001), respectively (mean AUC = 0.81). There was very good agreement between the two observers regarding feeding artery detection by 3D CT images (K = 0.829, *P* = 0.0001).

**Table 1:** Feeding artery detection by both observers using 3DCT and DSA images compared to Ground truth

		Ground truth	
		Positive (n = 173)	Negative (n = 12)
3D (observer-1/ Observer-2)	Positive	159/157	4/3
	Negative	14/16	8/9
DSA (observer-1/ Observer-2)	Positive	139 /136	5/5
	Negative	34/37	7/7
	3D (observer-1/observer-2)	DSA (observer-1/observer-2)	
Sensitivity	91.9% / 90.8%	80.3% / 78.6%	
Specificity	66.7% / 75%	58.3% / 58.3%	
PPV	97.5% / 98.1%	96.5% / 96.5%	
NPV	36.4% / 36%	17.1% / 15.9%	
Accuracy	90.3% / 89.7%	78.9% / 77.3%	

DSA detection of feeding arteries by observer1 versus observer2 revealed a sensitivity of 80.3% versus 78.6% (mean = 80%), a specificity of 58.3% versus 58.3% (mean = 58%), a PPV of 96.5% versus 96.5% (mean 96.5%), an NPV of 17.1% versus 15.9% (mean = 16.5%), and an accuracy of 78.9% versus 77.3% (mean = 78%). ROC curve analysis of the results for observers 1 and 2 revealed that the AUC was 0.69 (0.52–0.86, 95% CI; *P* = 0.025) and 0.69 (0.52–0.85, 95% CI; *P* = 0.03), respectively (mean AUC = 0.69). There was very good agreement between the two observers regarding feeding artery detection by DSA (k = 0.863, *P* = 0.0001).

For lesions ≤ 2 cm, DSA detection of feeding arteries by both observers revealed a mean sensitivity of 45%, a mean specificity of 50%, a mean PPV of 97%, a mean NPV of 2% and a mean accuracy of 45%, while 3DCT revealed a mean sensitivity of 89%, a mean specificity of 100%, a mean PPV of 100%, a mean NPV of 18.5%, and a mean accuracy of 89%.

**Fig. 3:**
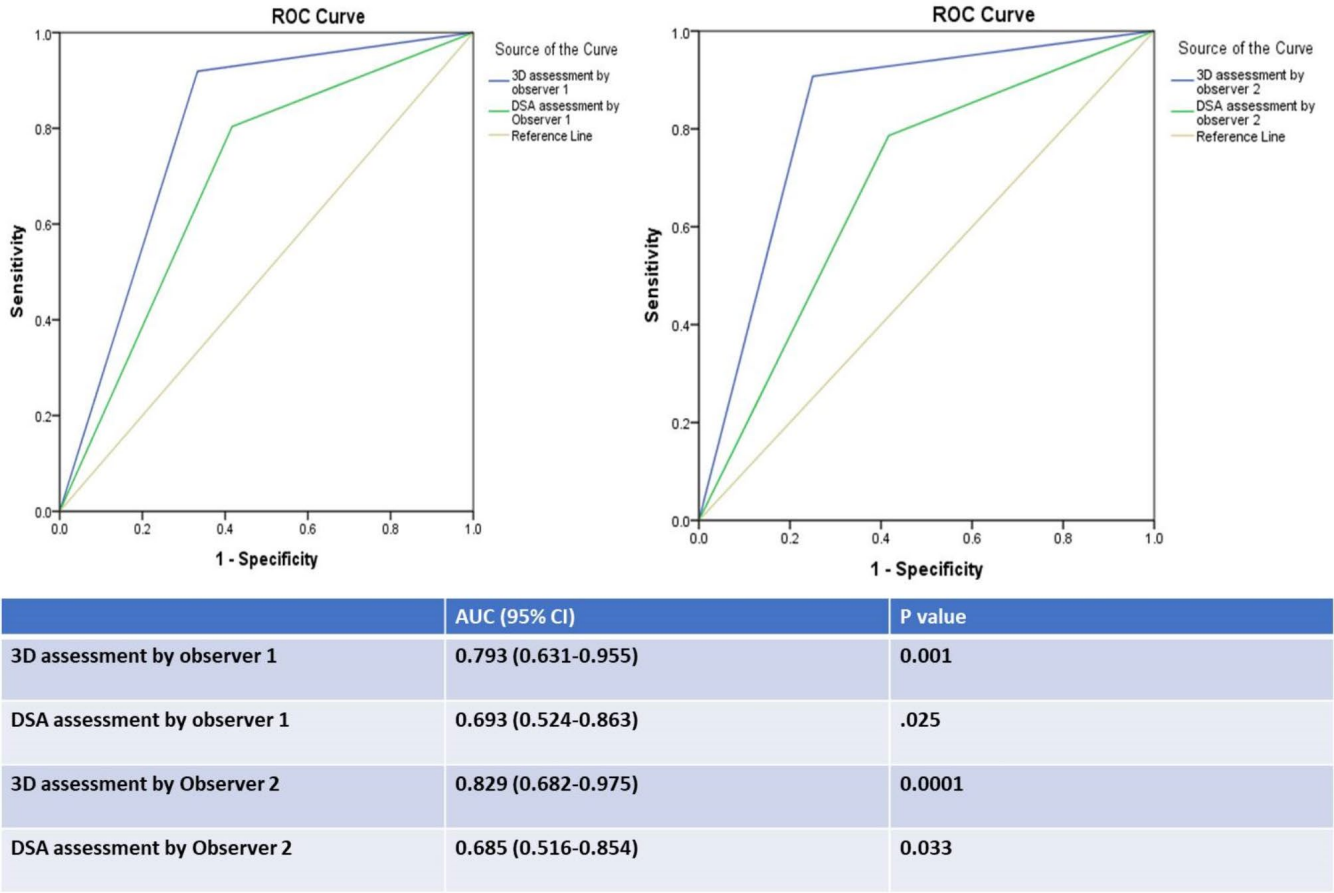
ROC curve of 3DCT and DSA detection of feeding arteries by both observers with AUC values

The accuracy of detection of the feeding arteries and the area under the receiver operating characteristic (ROC) curve (AUC) were greater for 3D CT than for DSA for both observers (Fig. 3).

Among the 136 detected active HCCs, the rate of 3DCT detection of all feeding arteries of each lesion was higher than DSA by both observers but without statistical significance i.e. observer1 has detected all feeding arteries of 124 lesions (91.2%) by CT and all feeding arteries of 107 lesions (76.5%) by DSA (X^2^ test, *P* = 0.15) while observer2 has detected all feeding arteries of 121 lesions (89%) by CT and all feeding arteries of 100 lesions (73.5%) by DSA (X^2^ test, *P* = 0.22).

### Factors affecting accuracy (Table 2)

There was a significant association between ***3D CT image quality and the accuracy*** of detection of the feeding vessels by both observers, as false negative results were greater for ***“Lobar "*** 3DCT image quality than for ***“segmental and subsegmental”*** image qualities (X^2^ test with post hoc analysis, *P* = 0.0001), and true positive results were lower for ***“Lobar”*** 3DCT image quality than for ***“segmental and subsegmental”*** image qualities (X^2^ test with post hoc analysis, *p* = 0.0001). However, there was no significant association between ***DSA quality and the accuracy*** of detection of feeding vessels by either observer (X^2^, *P* = 0.78).

**Table 2:** Association between detection of feeding arteries and lesion size and type categories and image quality

	True positive		False Positive		True Negative		False Negative	
	3D	DSA	3D	DSA	3D	DSA	3D	DSA
Observer1								
≤ 2 cm	36(87.8%)	**18(43.9%)**	0	0	1(2.4%)	1(2.4%)	4(9.8%)	**22(53.7%)**
> 2 cm	123(85.4%)	**121(84%)**	4(2.8%)	5(3.5%)	7(4.9%)	6(4.2%)	10(6.9%)	**12(8.3%)**
Observer2								
≤ 2 cm	35(85.4%)	**18 (43.9%)**	0	1(2.4%)	1(2.4%)	0	5(12.2%)	**22(53.7%)**
> 2 cm	122(84.7%)	**118(81.9%)**	3(2.1%)	4(2.8%)	8(5.6%)	7(4.9%)	11(7.6%)	**15(10.4%)**
	3D	DSA	3D	DSA	3D	DSA	3D	DSA
Observer1								
Capsule	**150(88.2%)**	126(74.1%)	4(2.4%)	5(2.9%)	7(4.1%)	6(3.5%)	**9(5.3%)**	33(19.4%)
Diffuse	**9(60%)**	13(86.7%)	0	0	1(6.7%)	1(6.7%)	**5(33.3%)**	1(6.7%)
Observer2								
Capsule	**149(87.6%)**	123(72.4%)	3(1.8%)	5(2.9%)	8(4.7%)	6(3.5%)	**10(5.9%)**	36(21.2%)
Diffuse	**8(53.3%)**	13(86.7%)	0	0	1(6.7%)	1(6.7%)	**6(40%)**	1(6.7%)
	3D	DSA	3D	DSA	3D	DSA	3D	DSA
Observer1								
Subseg.	**109(87.2%)**	112(74.2%)	4(3.2%)	5(3.3%)	6(4.8%)	7(4.6%)	**6(4.8%)**	27(17.9%)
Segment	**50(87.7%)**	26(78.8%	0	0	2(3.5%)	0	**5(8.8%)**	7(21.2%)
Lobar	**0**	1(100%)	0	0	0	0	**3(100%)**	0
Observer2								
Subseg.	**107(85.6%)**	109(72.2%)	3(2.4%)	5(3.3%)	7(5.6%)	7(4.6%)	**8(6.4%)**	30(19.9%)
Segment	**50(87.7%)**	26(78.8%)	0	0	2(3.5%)	0	**5(8.8%)**	7(21.2%)
Lobar	**0**	1(100%)	0	0	0	0	**3(100%)**	0

Bold numbers are values with statistically significant difference, *P* < 0.05

There was a significant association between the ***type of lesion and the 3D CT accuracy*** of feeding artery detection by both observers, i.e., true positive results were greater in capsulated lesions (X^2^ test with post hoc analysis, *P* = 0.003), and false negative results were lower in capsulated lesions (X^2^ test with post hoc analysis, *P* = 0.0001). There was no significant association between the ***type of lesion and DSA accuracy*** of detection of the feeding arteries by both observers (X^2^ test, *P* = 0.45).

There was a significant association between ***lesion size and DSA accuracy*** of feeding artery detection by both observers, i.e., true positivity was greater in lesions > 2 cm, while false negativity was greater in lesions ≤ 2 cm (X^2^ test with post hoc analysis, *P* = 0.0001). However, there was no association between ***size category and 3D CT accuracy*** of detection of feeding vessels by either observer (X^2^ test, *P* = 0.5).

In the 69 patients, DSA was done using 5Fr catheter in 60 patients (87%) and using microcatheter in 9 patients (13%). Among these 9 patients, the 5 Fr catheter could not be advanced in the ostium of the replaced right hepatic artery in 7 patients or in the celiac trunk in one patient with celiac stenosis and another patient with celiac occlusion. There were no statistically significant differences between the 5 Fr catheter DSA and microcatheter DSA in detecting feeding arteries as observed by both observers. For observer1, TP feeders were 75.3% (*n* = 122) for the 5 Fr catheter DSA and 73.9% (*n* = 17) for the microcatheter DSA, while FP feeders were 2.5% (*n* = 4) and 4.3% (*n* = 1), respectively. TN feeders were 3.1% (*n* = 5) and 8.7% (*n* = 2), and FN feeders were 19.1% (*n* = 31) and 13% (*n* = 3), (X^2^ test; *P* = 0.5). Observer1 missed three feeding arteries in DSA using the microcatheter, which were detected by 3D CT images. For observer2, TP feeders were 74.1% (*n* = 120) for the 5 Fr catheter DSA and 69.6% (*n* = 16) for the microcatheter DSA, while FP feeders were 2.5% (*n* = 4) and 4.3% (*n* = 1), respectively. TN feeders were 3.1% (*n* = 5) and 8.7% (*n* = 2), and FN feeders were 20.4% (*n* = 33) and 17.4% (*n* = 4), (X^2^ test, *P* = 0.55). Observer2 missed four feeding arteries in DSA using the microcatheter, which were detected by 3D CT images.

### The degree of confidence and the factors affecting it (Table 3)

The degree of confidence of feeding artery detection by 3D CT by observers 1 and 2 was ***“definitely feeding artery”*** in 106 versus 98 arteries, ***“probably feeding artery”*** in 26 versus 34, ***“intermediate probability”*** in 31 versus 28 and ***“not a feeding artery”*** in 22 versus 25. There was very good agreement between both observers regarding the degree of confidence in feeding artery detection by 3DCT (k = 0.828, *P* = 0.0001).

**Table 3:** Association between degree of confidence in feeding artery detection and lesion size category and image quality grades

	Definite	Probable	Intermediate probability	Not feeding A.	*P* value
**3D_observer1**	106 (57.3%)	26 (14.1%)	31(16.8%)	22(11.9%)	
***Lesion size*** >2 cm ≤ 2 cm	**101 (70.1%)** **5 (12.2%)**	16 (11.1%) 10 (24.4%)	**10 (6.9%)** **21 (51.2%)**	17 (11.8%) 5 (12.2%)	**0.0001**
***3D quality*** Subsegment Segmental Lobar	70 (56%) 36 (63.2%) 0	21 (16.8%) 5 (8.8%) 0	22 (17.6%) 9 (15.8%) 0	12(9.6%) 7 (12.3%) **3 (100%)**	**0.0001**
**DSA_observer1**	94(50.8%)	38 (20.5%)	12 (6.5%)	41 (22.2%)	
***Lesion size*** >2 cm ≤2 cm	**85 (59%)** **9 (22%)**	33 (22.9%) 5 (12.2%)	8 (5.6%) 4 (9.8%)	**18 (12.5%)** **23 (56.1%)**	**0.0001**
***DSA quality*** Subsegment Segmental Lobar	78 (51.7%) 16 (48.5%) 0	30 (19.9%) 7 (21.2%) 1(100%)	9(6%) 3 (9.1%) 0	34(22.5%) 7 (21.2%) 0	0.62
**3D_observer2**	98 (53%)	34 (18.4%)	28 (15.1%)	25 (13.5%)	
***Lesion size*** >2 cm ≤2 cm	**93 (64.6%)** **5 (12.2%)**	26 (18.1%) 8 (19.5%)	**6 (4.2%)** **22 (53.7%)**	19 (13.2%) 6 (14.6%)	**0.0001**
***3D quality*** Subsegment Segmental Lobar	65(52%) 33(57.9%) 0	26 (20.8%) 8 (14%) 0	19 (15.2%) 9 (15.8%) 0	15 (12%) 7 (12.3%) **3 (100%)**	**0.002**
**DSA_observer2**	100 (54.1%)	33 (17.8%)	8 (4.3%)	44 (23.8%)	
***Lesion size*** >2 cm ≤2 cm	**89 (61.8%)** **11 (26.8%)**	30 (20.8%) 3 (7.3%)	**3 (2.1%)** **5 (12.2%)**	**22 (15.3%)** **22 (53.7%)**	**0.0001**
***DSA quality*** Subsegment Segmental Lobar	84 (55.6%) 16 (48.5%) 0	23 (15.2%) 9 (27.3%) 1 (100%)	7 (4.6%) 1 (3%) 0	37 (24.5%) 7 (21.2%) 0	0.288

Bold numbers are values with statistically significant difference, *P* < 0.05

The degree of confidence of feeding artery detection by 3DCT by both observers as ***“definite feeding artery***” was significantly greater in lesions > 2 cm than in lesions ≤ 2 cm (X^2^ test with post hoc analysis, *p* = 0.0001), while ***“intermediate probability”*** was significantly lower in lesions > 2 cm than in lesions ≤ 2 cm (X^2^ test with post hoc analysis, *p* = 0.0001). The degree of confidence was also significantly lower in image quality, graded as ***“lobar”*** than ***“subsegmental and segmental”*** quality grades, for both observers (X^2^ test with post hoc analysis, *p* = 0.0001).

The degree of confidence of feeding artery detection by DSA by observers 1 and 2 was ***“definitely a feeding artery”*** in 94 versus 100 arteries, ***“probably a feeding artery”*** in 38 versus 33 arteries, ***“intermediate probability”*** in 12 versus 8 arteries and ***“not a feeding artery”*** in 41 versus 44 arteries, respectively. There was very good agreement between both observers regarding the degree of confidence in feeding artery detection by DSA (k = 0.846, *P* = 0.0001).

The degree of confidence in feeding artery detection by DSA as ***“definite feeding artery”*** by both observers was significantly greater in lesions > 2 cm than in lesions ≤ 2 cm, while the degree of not detecting the feeding artery or intermediate probability of being a feeding artery was significantly greater in lesions ≤ 2 cm than in lesions > 2 cm (X^2^ test with post hoc analysis, *P* = 0.0001). However, there was no significant difference in confidence score between the different image quality grades. (X^2^test, *P* = 0.62 and 0.28 for both observers).

## Discussion

HCC is the sixth most common cancer globally [1] and TACE is the primary standard treatment for intermediate-stage HCC according to the BCLC staging system [13]. Accurate assessment of tumor-feeding arteries is essential for successful TACE while minimizing liver damage. The sensitivities of non-selective DSAs for detecting feeding vessels were reported to be 38% [15], 39.6% [4], 54.4% [16], 64% [8], 71.8% [17], and 77.2% [18]. Our study demonstrated a higher sensitivity (79.5%) of non-selective DSA in detecting feeding arteries compared to previous reports, which can be attributed to larger lesion sizes in our study. We found that lesions > 2 cm had higher true positivity rates, while lesions ≤ 2 cm had higher false negativity rates. Hence, larger lesions improve DSA accuracy.

**Fig. 4:**
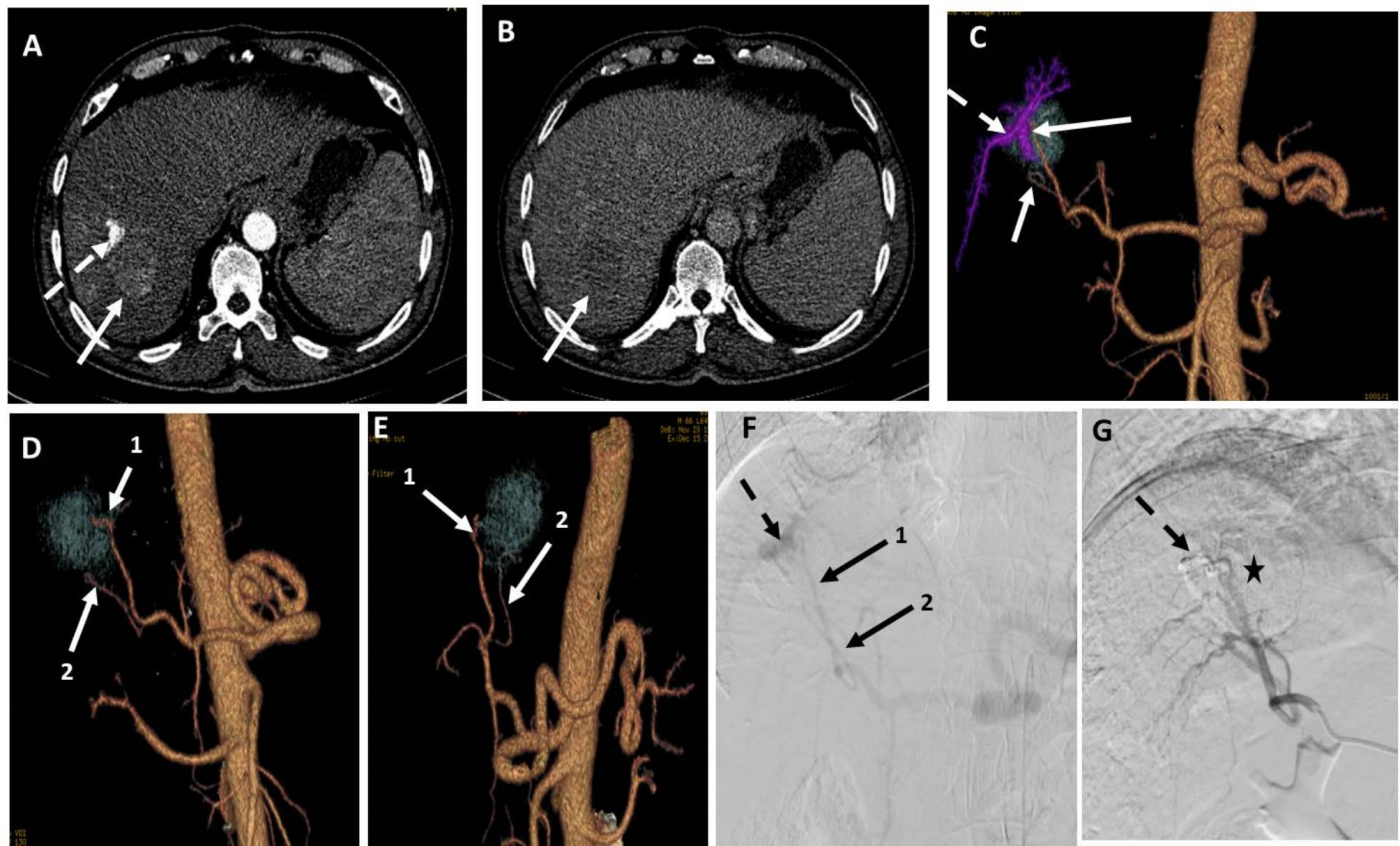
A patient with segment VII HCC measuring 4 cm. (**A**) and (**B**) are axial CT images in the arterial phase and venous phase, respectively, showing early arterial contrast enhancement of the lesion with washout in the venous phase (arrows). Noted arterio-portal shunt in the arterial phase (dashed arrow). (**C**) 3D reconstructed AP image showing the arterioportal shunt, which is coded in blue (dashed arrow), and two possible feeding arteries. (**D**) and (**E**) are 3D-reconstructed images in oblique views after subtraction of the arterioportal fistula showing the two possible feeding arteries clearly. (**F**) DSA image obtained using a 5fr catheter in the celiac trunk showing an arterioportal fistula (dashed arrow) and two possible feeding arteries. (**G**) DSA image of the right hepatic artery using a 5Fr catheter after embolization of the arterioportal fistula with coils and injection of chemoembolization agents with lipiodol entrapment in the lesion (asterisk). Both observers identified artery 1 in both 3D images and DSA images, but artery 2 was only identified in 3D images by both observers. Both arteries were true feeders according to ground truth data

The limitations of DSA in detecting feeding arteries stem from its 2D nature, which makes it challenging to discern precise relationships between overlapping vessels. Multiple acquisitions and catheter positions are often required, leading to increased radiation exposure and contrast usage. Additionally, DSA may miss tumours due to hypovascularity or irregular enhancement patterns in cirrhotic liver parenchyma [19]. Miyayama et al. and Ushijima et al. [4, 15] reported that only 72.1-79.5% of HCCs could be visualized by non-selective DSA. Differentiating hypervascular non-tumorous staining areas from true lesions using non-selective DSA is also challenging [20]. In our study, we encountered difficulties visualizing lesion enhancement during non-selective DSA due to presence of an arterio-portal shunt and occlusion of the shunt was performed before completing TACE **(**Fig. 4**).**

Additionally, the technical difficulty of obtaining good-quality DSAs increases when vascular anatomical variations or potential stenoses or occlusions in the celiac or hepatic arteries are encountered. In our study, we faced a patient with complete celiac artery-origin occlusion with a dilated pancreatico-duodenal arcade. In this case, the TACE procedure was terminated due to difficulty in catheterizing the pancreatico-duodenal arcade from the SMA and a second TACE procedure was performed successfully after using 3D VR images as reference images to catheterize the best dilated less tortuous collateral route.

Multidetector computed tomography (MDCT) scanners offer improved temporal and spatial resolutions, allowing for dynamic imaging of HCC [9]. The shorter acquisition time of MDCT allows for the acquisition of pure arterial angiographic images with the ability to visualize even the fine distal branches of the hepatic arteries [21]. Advancements in 3D hardware and software technology have significantly improved the quality of 3D rendering images that can be obtained from routine CT scans performed for tumor detection, eliminating the need for additional contrast material or radiation. These images were proven to be very useful for evaluating hepatic artery anatomy before hepatic tumor resection [22] or liver transplantation [23].

In a study conducted by Kim et al. [12], the accuracy of detecting subsegmental feeding vessels was reported to be 22.2% with VR images and 77.8% with MIP images. MIP images were found to have good contrast between the hepatic parenchyma, hepatic arteries, and HCC, leading to better feeding vessel detection. On the other hand, it is sometimes difficult to distinguish small intrahepatic arteries from contrast-enhanced hepatic parenchyma in VR images. In our study, we used a specific technique for generating 3D VR images in which the arteries were selected but the hepatic and soft tissue backgrounds were removed. After that, a separate image of the tumor was added to the 3D VR arterial image. This technique improved the detectability of tumor-feeding vessels compared to that in Kim et al.‘s study. Minami et al [16] reported that 3D CT imaging was significantly better for accurate detection of feeding arteries than 2D celiac angiography, with a sensitivity of 97.1% versus 54.4%, a specificity of 80% versus 60% and an accuracy of 90.4% versus 50.7%, respectively. Our results are consistent with Minami et al. results. Additionally, these 3D VR images help in freely determining the most favourable working angle to isolate the feeding arteries from other overlapping arteries and can be used as a reference image during TACE. Some Experts use MIP images of variable thickness (e.g. 5–100 mm) in coronal plane then make additional angulation of the plane and windowing to detect feeders and then use this angulation as a working angle of the C-arm in the angio-lab. Capsulated lesions and good-quality 3D images are associated with improved feeding artery detection by 3D CT, while lesion size affects only the degree of confidence in feeding artery detection, as shown in our results.

**Fig. 5:**
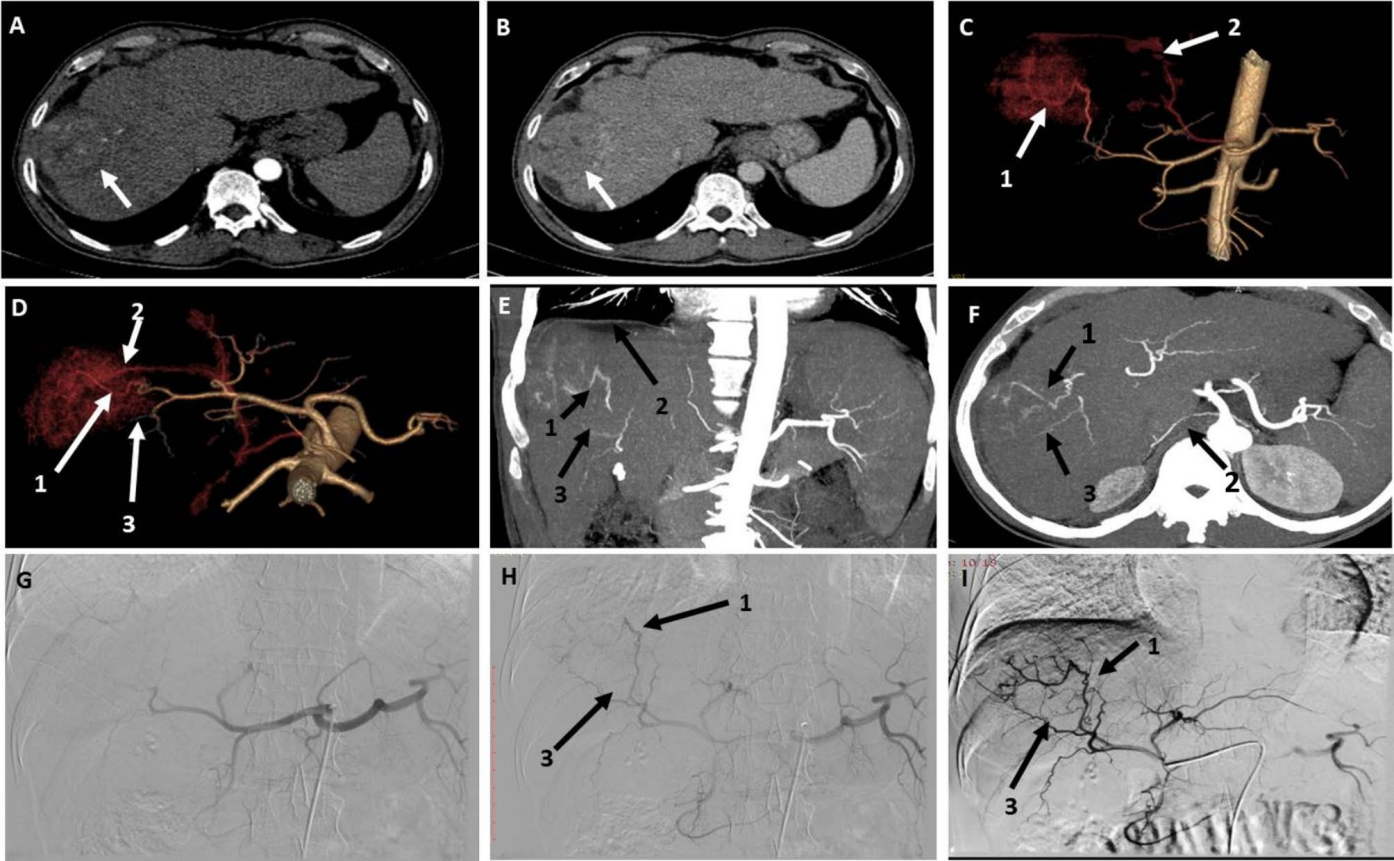
A patient with segment VII HCC measuring 6 × 5 cm. (**A**) and (**B**) Axial CT images in the arterial and portal phases, respectively, showing early arterial enhancement of the lesion with early contrast washout. (**C**) and (**D**) are 3D reconstructed angiography images in the AP view and cranio-caudal view, respectively, showing three possible feeding arteries numbered 1, 2, and 3. (**E**) and (**F**) Coronal and axial MIP CT images showing the three possible feeding arteries and the origin of artery no. 2 (right inferior phrenic artery) from the origin of the celiac trunk. (**G**) DSA of the celiac trunk using a 5fr catheter showing branches of the celiac trunk with no detectable contrast opacification of the right subphrenic artery (feeding artery no. 2). (**H**) and (**I**) DSA of the Celiac artery and CHA using a 5fr catheter showing the other two possible feeding arteries 1 and 3. Observer 1 detected arteries 1, 2 and 3 in 3D images, while observer 2 detected only arteries 1 and 3. Both observers identified arteries 1 and 3 in the DSA images. The three arteries are true feeding arteries for the lesion according to the ground truth

Extra-hepatic parasitic feeding vessels have been shown to complicate retreatment and affect the therapeutic outcomes of TACE [24]. These vessels can be missed during TACE, and thin-section CT has been reported to detect extrahepatic tumor feeders from the right inferior phrenic artery, internal mammary artery, and intercostal arteries in 56%, 79% and 54% of HCC patients, respectively [25–27]. The occurrence of these vessels increases with the size of the hepatic mass > 5 cm, involvement of the liver capsule and tumor location, particularly beneath the diaphragm or in the bare area of the liver [26–28]. In our study, one patient had an extra-hepatic feeding artery from the right inferior phrenic artery detected on 3D VR images but missed on DSA because the interventional radiologist did not perform selective phrenic artery angiogram and the tumor was mainly supplied by hepatic arteries from celiac trunk so the operator did not suspect the presence of extrahepatic supply for this case (Fig. 5).

CBCT provides three-dimensional images and allows production of DSA and CT-like soft-tissue images using the same device and has its advantages and limitations. It has high spatial resolution making it useful for observing thin subsegmental hepatic arteries [29] but it has lower contrast resolution compared to MDCT mainly due to increased beam scatter. CBCT can better identify tumor-feeding arteries than DSA with sensitivity range between 73 and 96.9% [8, 15, 16, 18]. Moreover, 3D and MIP images can be obtained from CBCT images and used as reference images during TACE and the best projection and angulation of the C-arm can be determined accurately and used for best visualization of the origin of the feeding artery facilitating its catheterization by microcatheter. Challenges with CBCT include small field of view, motion artifacts, image quality, lengthy analysis, and radiation dose [18].

A combined CT and angiography system (Angio-CT) have been used to offer excellent intraprocedural tumor and vascular depiction [12] but associated costs and workflow considerations have limited their widespread adoption. Angio-CT has improved the detection of feeding arteries, including extrahepatic ones, but it requires multiple DSA and CT examinations, leading to increased radiation exposure and contrast medium use [30].

2D-3D registration techniques are developed to integrate pre/perioperative 3D vasculature data with intraoperative 2D X-ray images offering a real-time roadmap to improve procedural guidance and reduce intervention time, radiation exposure, and contrast agent use. Challenges in abdominal interventions due to respiratory motion have led to developing methods like tracking moving regions of interest for registration. A catheter-based registration method is reported to enhance accuracy by matching vessel centre-lines from 3D images with the 2D catheter shape (10).

Limitations of our study include; (a) Manual contrast injection during DSA, which may affect the detection of small feeding vessels, (b) The use of CT data from different centres and machines with varying protocols. Generating clear 3D VR images using our technique needs a high-quality workstation software and a well-trained technician. We can confirm that ADVANTAGE Workstation from GE company and SYNAPSE VINCENT workstation from Fujifilm company are suitable softwares to generate these multiple separate 3D images that can be merged after that as desired using our described technique. Also obtaining these clear 3D VR images takes about 5 to 15 min according to technician experience and complexity of vascular anatomy. Hence, the variability in the availability and implementation of 3D CT angiography technology and the potential variability in imaging quality across different institutions could affect generalization of this study results. So, further studies on other available workstations and across different clinical settings and patient populations should be considered. C) Another limitation of the study is the use of Ground truth as a gold standard because CBCT, which is considered as the gold standard by experts’ consensus [31], was not done in all cases.

## Conclusions

3D VR CT angiography images have shown promise in improving the detection of HCC feeding vessels compared to DSA and can be useful for TACE planning or used as reference images during TACE which may reduce the procedure time, radiation and contrast used and increase TACE efficacy. However, further studies are required to confirm these findings across different clinical settings and patient populations.

## Electronic supplementary material

Below is the link to the electronic supplementary material.

Supplementary Material 1

Supplementary Material 2

Supplementary Material 3

## Data Availability

The datasets used and/or analyzed during the current study available from the corresponding author on reasonable request.

## Abbreviations

2D: Two-dimensional
3D: Three-dimensional
Angio-CT: Combined CT and angiography
AUC: Area under the curve
CB-CT: Cone-beam computed tomography
CHA: Common hepatic artery
DSA: Digital subtraction angiography
FN: False negative
FP: False Positive
GT: Ground truth
HCC: Hepatocellular carcinoma
MDCT: Multidetector CT
MIP: Maximum intensity projection
NPV: Negative predictive value
PHA: Hepatic artery proper
PPV: Positive predictive value
SMA: Superior mesenteric artery
TACE: Transcatheter arterial chemoembolization
TN: True negative
TP: True positive
VR: Volume rendering
X^2^: Chi-square test
