# Supplementary material for: Detection of hepatocellular carcinoma feeding vessels: MDCT angiography with 3D reconstruction versus digital subtraction angiography
Source: BMC Med Imaging. 2024 Sep 18;24:250. doi: 10.1186/s12880-024-01408-z (PMC11412056; doi:10.1186/s12880-024-01408-z)
Supplement: Supplementary file 3 — Supplementary Material 3 [file 12880_2024_1408_MOESM3_ESM.docx]

**Supplementary files legend**

**Supplementary file 1 and 2.MP4 :**

Video files showing the technique of obtaining 3D VR images from arterial and portal phases of pre-TACE multiphasic CT in a patient with segment VII HCC.
